# Genome-Wide Identification of MADS-Box Genes in *Taraxacum kok-saghyz* and *Taraxacum mongolicum*: Evolutionary Mechanisms, Conserved Functions and New Functions Related to Natural Rubber Yield Formation

**DOI:** 10.3390/ijms241310997

**Published:** 2023-07-01

**Authors:** Jiaqi Chen, Yushuang Yang, Chuang Li, Qiuhui Chen, Shizhong Liu, Bi Qin

**Affiliations:** 1 Key Laboratory of Biology and Genetic Resources of Rubber Tree, Ministry of Agriculture and Rural Affairs, Rubber Research Institute, Chinese Academy of Tropical Agricultural Sciences, Haikou 571101, China; 2 Institute of Tropical Crops, Hainan University, Haikou 570228, China

**Keywords:** MADS-box gene, *Taraxacum kok-saghyz*, *Taraxacum mongolicum*, collinearity, biomass, natural rubber biosynthesis

## Abstract

MADS-box transcription regulators play important roles in plant growth and development. However, very few MADS-box genes have been isolated in the genus *Taraxacum*, which consists of more than 3000 species. To explore their functions in the promising natural rubber (NR)-producing plant *Taraxacum kok-saghyz* (TKS), MADS-box genes were identified in the genome of TKS and the related species *Taraxacum mongolicum* (TM; non-NR-producing) via genome-wide screening. In total, 66 *TkMADSs* and 59 *TmMADSs* were identified in the TKS and TM genomes, respectively. From diploid TKS to triploid TM, the total number of MADS-box genes did not increase, but expansion occurred in specific subfamilies. Between the two genomes, a total of 11 duplications, which promoted the expansion of MADS-box genes, were identified in the two species. *TkMADS* and *TmMADS* were highly conserved, and showed good collinearity. Furthermore, most *TkMADS* genes exhibiting tissue-specific expression patterns, especially genes associated with the ABCDE model, were preferentially expressed in the flowers, suggesting their conserved and dominant functions in flower development in TKS. Moreover, by comparing the transcriptomes of different TKS lines, we identified 25 *TkMADSs* related to biomass formation and 4 *TkMADSs* related to NR content, which represented new targets for improving the NR yield of TKS.

## 1. Introduction

MADS-box gene families are widespread in plants, animals, and fungi. MADS-box transcription factors share a highly conserved MADS domain consisting of 56–58 amino acids [1]. Based on their protein structure, MADS-box transcription factors can be classified into two types, type I and type II [2]. Type I proteins have only a MADS domain, and can be further classified into three subclasses, i.e., Mα, Mβ, and Mγ [3]. Type II genes include Myocyte enhancer factor 2 (MEF2)-like genes in animals and fungi, and MIKC-type genes in plants; the products of these genes contain a MADS (M) domain, an intervening (I) domain, a keratin-like (K) domain, and a C-terminal (C) domain [4]. The conserved MADS domain is responsible for the DNA binding, nuclear localization, and dimerization of proteins. The I and K domains are essential for the dimerization and interactions of proteins, while the C domain is highly variable and is required for transcriptional activation [3,4]. Based on phylogenetic relationships and structural features, plant MIKC genes can be further subdivided into the MIKC^C^ and MIKC* types, and the MIKC^C^ type can be further divided into fourteen distinct subfamilies [5]. With the development of high-throughput sequencing technology, a large number of MADS-box transcription factor-encoding genes have been identified in a wide variety of plant species, including the model plant species *Arabidopsis* [6] and rice [7] and nonmodel plant species including wheat [5], chrysanthemum [8], watermelon [9], tomato [10], etc.

MADS-box genes play important regulatory roles in plant growth, development, and stress responses [5,7,11,12,13,14]. These genes are ubiquitous in the plant kingdom, and they are expressed at different stages of development and in different organs [15,16,17]. The functions of type II genes, especially MIKC^C^-type MADS-box genes, have been reported in plants, but only a few type I genes have been identified. Several MADS-box genes may serve as important components of gene regulatory networks (GRNs), thus forming the basis of the stem cell microenvironment [18]. MADS-box genes are involved in the well-known ABCDE model of flower development. The A-class genes, *APETALA1* (*AP1*) and *AP2*, determine sepal identity [19]. The B-class genes, *AP3* and *PISTILLATA* (*PI*) combined with the A-class genes control petal identity, while the C-class gene *AGAMOUS* (*AG*) and B-class genes regulate stamen identity, and the C-class gene alone determines carpel formation [20,21,22]. D-class genes *SHATTERPROOF 1* (*SHP1*), *SHP2*, and *SEEDSTICK* (*STK*) specify ovule identity and differentiation [23]. Among the flowering-related MADS-domain proteins, the E-class proteins, which include the four largely redundantly acting SEP subfamily members (SEP1-4), have specific roles as mediators of higher-order complex formation [24]. Although *Taraxacum* plants have diverse reproduction characteristics (including apomixis, self-incompatibility, and self-compatibility), no MADS-box genes regulating floral development have been illustrated in *Taraxacum* plants.

In addition, there is evidence that MADS-box genes regulate root growth and development. For example, the MADS-box gene *SRD1* regulates the formation and development of storage roots in sweet potato [25]. A recent study also indicated that MADS-box genes were involved in the regulation of natural rubber (NR) biosynthesis. Rubber tree HblMADS24 binds to the promoter of *HbFPS1*, a key enzyme-encoding gene involved in NR biosynthesis, and transcriptionally activates the *HbFPS1* promoter, suggesting that HblMADS24 is a transcriptional activator of *HbFPS1* [26]. HbMADS4 binds with the promoter of *HbSRPP*, and transient overexpression of HbMADS4 in tobacco was shown to repress the activity of the *HbSRPP* promoter significantly [27]. However, the exact regulatory mechanism of the two MADS-box genes in NR biosynthesis is still unknown. *Taraxacum kok-saghyz* (TKS) as a promising alternative rubber-producing species, and *Taraxacum mongolicum* (TM) belong to the genus *Taraxacum*, which comprises more than 3000 species that can synthesize NR, inulin, dandelion sterol, taraxacerin, choline, and other important metabolites. Although TKS and TM belong to the same genus (*Taraxacum*), their metabolites and reproduction systems are completely different [28]. TM, an agamospermous triploid (2n = 24), is an excellent plant resource that has the concomitant functions related to both medicine and foodstuffs, but rarely produces NR in the roots. TKS, which is self-incompatible and diploid (2n = 16), produces high-quality NR in its roots, which makes it a promising NR-producing plant and an ideal model plant for studying NR biosynthesis [29]. However, because of the low biomass and low NR content, extracting NR from TKS roots is very expensive. In addition, self-incompatibility characteristics make it difficult to preserve the excellent traits of TKS varieties, but these disadvantages limit the commercial planting of TKS. Although the important regulatory functions of MADS-box genes in flower development, root morphogenesis, and NR biosynthesis have been reported, very few MADS-box genes in the *Taraxacum* genome have been identified, which limits the application of the MADS-box genes in the domestication and genetic improvement of TKS. Therefore, members of the MADS-box gene family in TKS and TM were identified through a comprehensive analysis of the available transcriptomic and genomic data, and the phylogenetic classification, chromosomal distribution, and collinearity features were analyzed in this study. Then, a comparative analysis of the MADS-box genes between TKS and TM will make it easier to reveal the evolutionary mechanism of MADS-box genes and identify the genes specific to the TKS and TM genome. Moreover, genes related to NR yield formation in TKS were identified, which represented new targets for increasing the NR yield of TKS via transgenic and gene editing technologies.

## 2. Results

### 2.1. Identification of Taraxacum MADS-Box Genes in the Genomes of TKS and TM

To comprehensively identify MADS-box genes in TKS and TM, we screened the published genomic data via BLAST and HMMER searches. Moreover, the *Arabidopsis* MADS-box protein sequences were also used as queries to perform BLASTP searches against the TKS and TM genomic databases. Then, genes more than 99% identical or at the same physical location were considered the same gene. Finally, 66 nonredundant MADS-box genes with full-length open reading frames (ORFs) were identified from the TKS genome and named *TkMADS-1* to *TkMADS-66* (Supplemental Table S1). Similarly, 59 nonredundant MADS-box genes were identified from the TM genome and named *TmMADS-1* to *TmMADS-59* (Supplemental Table S2). The sequence length of the TkMADS proteins ranged from 100 amino acids (TkMADS-54) to 299 amino acids (TkMADS-56), the MW ranged from 11.54 kDa (TkMADS-54) to 37.03 kDa (TkMADS-49), and the pI ranged from 4.52 (TkMADS-30) to 11.20 (TkMADS-31). For the TmMADS proteins, their length ranged from 100 amino acids (TmMADS-25) to 360 amino acids (TmMADS-50), the MW ranged from 11.58 kDa (TmMADS-25) to 40.96 kDa (TmMADS-50), and the pI ranged from 4.49 (TmMADS-26) to 10.73 (TmMADS-25). All the TkMADS and TmMADS proteins contained a MADS domain or a K-box domain, and 31 TkMADSs and 34 TmMADSs contained both domains. These results indicated that the total number of MADS genes was not amplified from diploid TKS to triploid TM.

### 2.2. Phylogenetic Analysis and Classification of TkMADS and TmMADS Proteins

**Figure 1:**
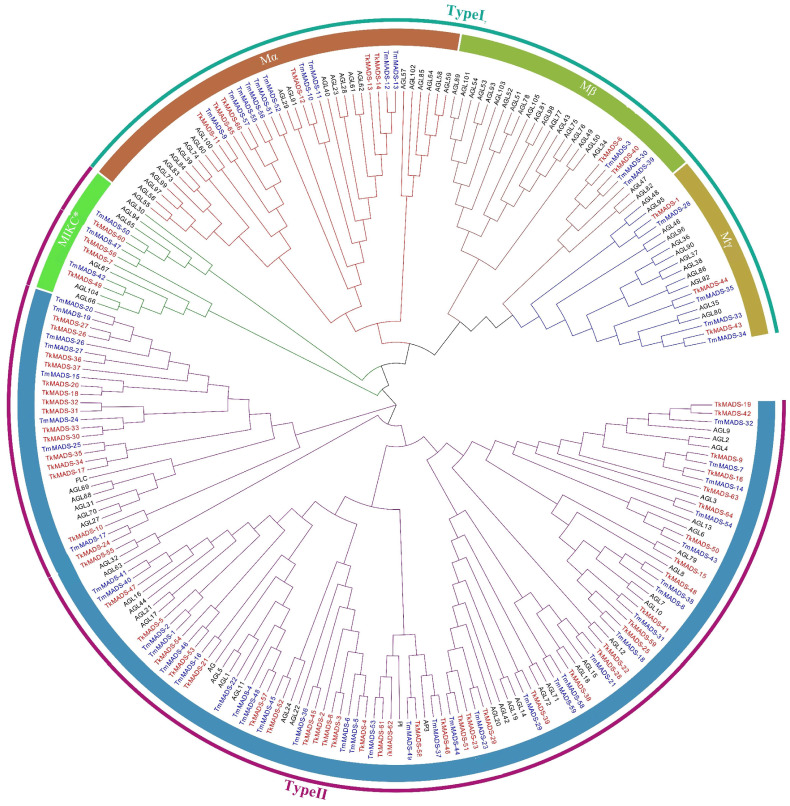
Phylogenetic relationships of *Taraxacum* and *Arabidopsis* MADS-box transcription factors. Phylogenetic analyses were conducted on MADS-box transcription factors from *Arabidopsis thaliana* (black), *Taraxacum kok-saghyz* (TKS, red), and *Taraxacum mongolicum* (TM, blue). ClustalW was used for multiple sequence alignment. The phylogenetic tree was constructed using the neighbor-joining (NJ) method with 1000 bootstrap repeats. Type I proteins are shown in cyan, and type II proteins are shown in purple.

**Figure 2:**
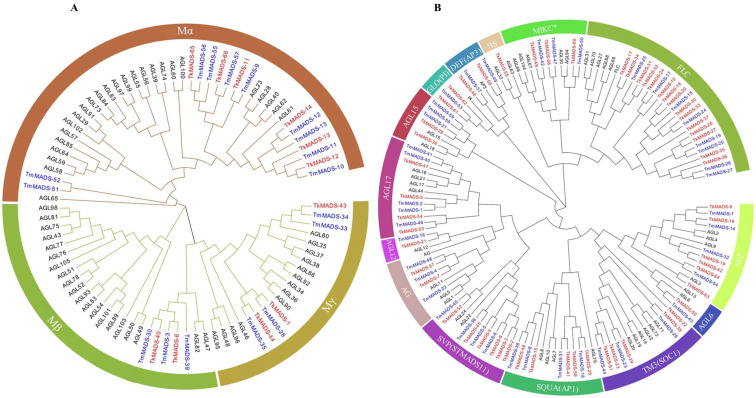
Phylogenetic analyses of the type I and type II MADS-box transcription factors. Phylogenetic analyses were performed on the type I (**A**) and type II (**B**) MADS-box transcription factors from *Arabidopsis thaliana* (black), TKS (red), and TM (blue). ClustalW was used for multiple sequence alignment. The phylogenetic tree was constructed using the NJ method with 1000 bootstrap repeats. Each subclade is represented by a specific color.

To investigate the phylogenetic relationships among TkMADS, TmMADS, and *Arabidopsis* MADS-box proteins [6], a phylogenetic tree was generated using MEGA X based on the neighbor-joining (NJ) method. As shown in Figure 1, 66 TkMADSs and 59 TmMADSs were divided into two types: type I and type II. Type I contained 11 TkMADS and 17 TmMADS proteins, and type II contained 55 TkMADS and 42 TmMADS proteins. According to the M domain of the MADS-box proteins, type I members can be further divided into three subgroups, namely, Mα, Mβ, and Mγ (Figure 2A). The Mα subfamily included 6 TkMADS and 10 TmMADS members, the Mβ subfamily included 2 TkMADS and 3 TmMADS members, and the Mγ subfamily included 3 TkMADS and 4 TmMADS members (Figure 2A). Type II genes can be further divided into the MIKC* and MIKC^C^ subgroups. Among the MIKC* subgroup, there were three members of both TkMADSs and TmMADSs. Furthermore, the MIKC^C^-type proteins were subdivided into 13 subfamilies: FLC, SEP, DEF (AP3), GLO (PI), AGL15, AGL17, AGL12, AG, STMADS11 (SVP), SQUA (AP1), TM3 (SOC1), AGL6, and BS (Figure 2B). TkMADS members were distributed in all 13 subfamilies, and TmMADS members were present in 12 subfamilies (the BS subfamily was excluded). Among them, FLC was the largest subfamily, consisting of 15 TkMADS and 8 TmMADS members, which was larger than that in *Arabidopsis*. Five subfamilies, SVP, SEP, SOC1, SQUA, and AGL17, were more abundant in TKS (6 SVP, 6 SEP, 5 SOC1, 5 SQUA, 4 AGL17) than in TM (4 SVP, 4 SEP, 3 SOC1, 4 SQUA, 5 AGL17). Each of the four subfamilies, AP3, PI, AGL15, and AG, contained two members of TkMADS, while there were 2 AP3, 1 PI, 3 AGL15, and 3 AG members of TmMADS. Both the AGL12 and AGL6 subfamilies contained only one member of *Taraxacum* (TKS and TM), and one TkMADS was present in the BS subfamily, which was absent in TM (Figure 2B). These results demonstrated that from diploid TKS to triploid TM, although the total number of MADS-box genes did not increase, specific subfamily genes showed significant expansion, such as the Mα subfamily in the TM genome and the FLC subfamily in the TKS genome.

### 2.3. Gene Structure and Conserved Motif Analysis of Taraxacum MADS-Box Genes

**Figure 3:**
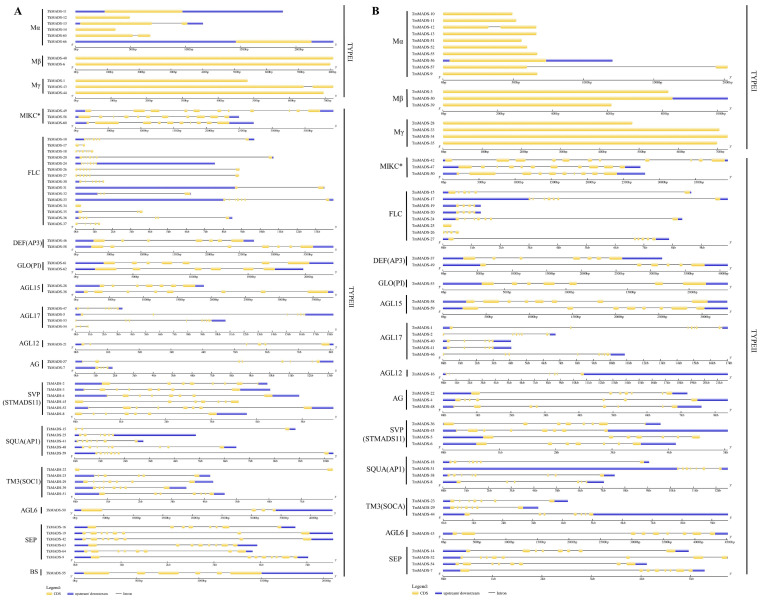
Gene structures of *TkMADS* and *TmMADS*. Analyses of gene structures of *TkMADS* (**A**) and *TmMADS* (**B**). Exon–intron structure analyses were performed using the Gene Structure Display Server (GSDS) database. The lengths of the exons and introns of each gene are shown on the scale of lines. The blue areas represent the UTR, the yellow areas represent the exons, and the black lines represent the introns.

To reveal the structural diversity of *Taraxacum* MADS-box genes, the intron/exon splicing was analyzed via the Gene Structure Display Server (GSDS) program by comparing the coding DNA sequence (CDS) of each gene with its genomic DNA sequence. As shown in Figure 3, the gene structures of *TkMADS* and *TmMADS* members belonging to the same subgroup were similar, but the type I and type II genes differed. The intron number of *Taraxacum* MADS-box genes ranged from 0 to 10. Most type I *TkMADS* and *TmMADS* genes had no introns; however, three *TkMADS* and two *TmMADS* genes contained one intron. The intron/exon structures of the type II genes were more complex than those of the type I genes. There were more than three introns in the type II genes, except that *TkMADS-34* and *TmMADS-25* had no introns. In addition, the highly homologous genes of the same subclade presented similar intron/exon splicing features, and these genes differed only in the length of introns and exons. However, some genes of the same subgroup exhibited different intron/exon arrangements. For example, *TkMADS-53* had eight exons, whereas its homolog *TkMADS-54* had only four exons; nonetheless, they showed a high degree of similarity in phylogenetic relationships.

**Figure 4:**
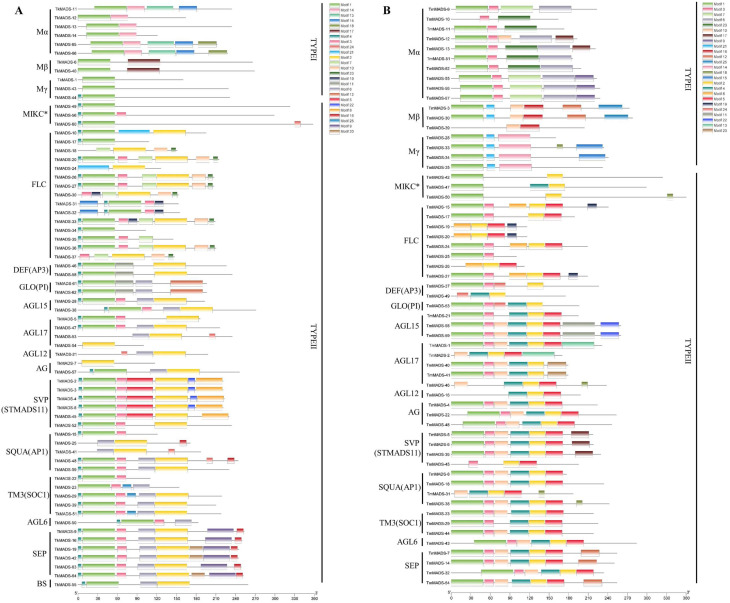
Conserved motifs of TkMADS and TmMADS proteins. Analyses of conserved motifs of TkMADS (**A**) and TmMADS (**B**) proteins. The conserved motifs were identified using the MEME database and are indicated by different colors. Motif 1 and motif 2 represent the MADS domain and K domain, respectively.

To clarify the conserved motifs of TkMADS and TmMADS proteins, the MEME program was used to analyze the conserved motifs, and the SMART online tool was used to annotate them. The results showed that a total of 25 conserved structural domains, named motifs 1 to 25, were identified in the TkMADS and TmMADS proteins (Figure 4 and Supplemental Table S3). Proteins of the same subclade contained the same motifs. Motif 1, which consists of 42 amino acids, represents the most typical MADS-box domain. Motif 1 was present in 55 (83%) TkMADS and 47 (80%) TmMADS proteins. Motif 2, motif 3, motif 4, and motif 6 were also conserved and present only in the type II proteins. Motif 2 and motif 4, which represent the K domain and play an important role in protein–protein interactions of MADS-box proteins, were found only in type II MADS-box proteins. Among the type II proteins, 47 TkMADS and 41 TmMADS members contained motif 2, and 44 TkMADS and 30 TmMADS members contained motif 4. However, some motifs (such as motif 13, motif 14, motif 15, motif 17, and motif 18) were poorly conserved and present only in type I proteins.

### 2.4. Chromosomal Localization of TkMADSs and TmMADSs in the Genome

**Figure 5:**
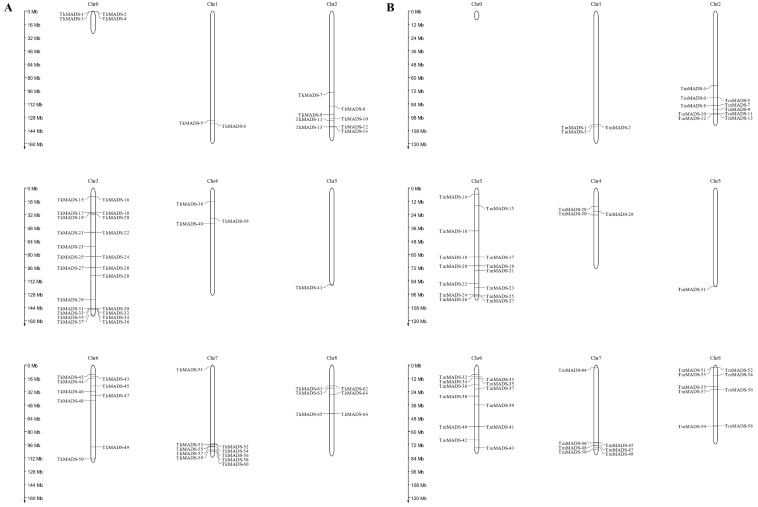
Chromosomal locations of MADS-box genes in the genomes of TKS and TM. Physical mapping of the *TkMADS* (**A**) and *TmMADS* (**B**) genes was conducted via MapChart based on the genomic data of TKS and TM. Eight chromosomes of TKS and TM are numbered Chr1~8, and Chr0 is a pseudochromosome that failed to assemble with the other eight chromosomes [28].

Based on the location of 66 *TkMADS* genes in the TKS genome and 59 *TmMADS* genes in the TM genome, their physical location on the chromosomes was mapped using MapChart. The results showed that *Taraxacum* MADS-box genes were unevenly distributed across eight chromosomes of TKS and TM (Figure 5). In the TKS genome, chromosome 3 contained the most *TkMADS* genes, followed by chromosomes 7, 6, 2, 8, 4, and 1, with 23, 10, 9, 8, 6, 3, and 2 genes, respectively. In the TM genome, the three chromosomes with most genes were chromosomes 3, 6, and 2, with 14, 12, and 10 *TmMADS* genes, respectively. Moreover, 9, 7, 3, and 3 *TmMADS* genes were mapped onto TM chromosomes 8, 7, 4, and 1, respectively. Both TKS and TM chromosome 5 contained the fewest MADS-box genes, each with only one—*TkMADS41* and *TmMADS31*, respectively. In addition, there were four *TkMADS* genes mapped onto the pseudochromosome chromosome 0, which failed to assemble with the other eight chromosomes [28]. Taken together, these results indicated that gene clusters were present at the chromosome ends, and both TKS and TM chromosomes 3 and 7 contained MADS-box gene clusters or gene hotspots.

### 2.5. Duplication and Synteny Analysis of Taraxacum MADS-Box Genes in the TKS and TM Genomes

**Figure 6:**
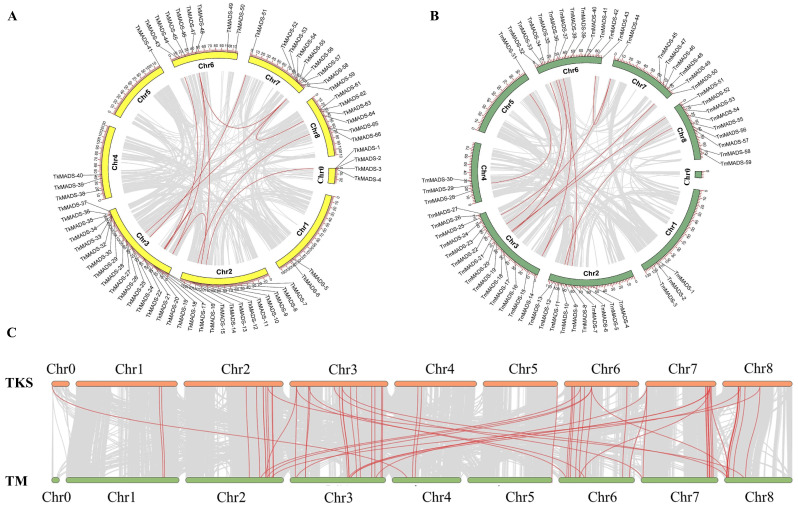
Duplication and synteny analysis of *TkMADS* and *TmMADS* genes. The red lines indicate duplications within the genomes of TKS (**A**) and TM (**B**). (**C**) All the collinear *TkMADS* and *TmMADS* genes are shown in red lines in the genomes of TKS and TM. The grey lines indicate collinear genes.

Gene duplication events play an important role in amplifying the number of gene families and increasing the genome complexity in eukaryotes, and tandem duplication or fragmental duplication of chromosomal regions usually leads to the expansion of gene family members [30,31]. Therefore, the duplication events of each *TkMADS* and *TmMADS* gene were analyzed using MCScanX. As shown in Figure 6, eleven duplications were identified in the TKS genome, involving 17 *TkMADS* genes (*TkMADS-2, TkMADS-8, TkMADS-9, TkMADS-11, TkMADS-15, TkMADS-16, TkMADS-17, TkMADS-19, TkMADS-20, TkMADS-23, TkMADS-25, TkMADS-42, TkMADS-48, TkMADS-51, TkMADS-59, TkMADS-63,* and *TkMADS-65*) (Figure 6A and Table 1). In the TM genome, the *TmMADS* gene family also experienced 11 duplication events involving 15 members (*TmMADS-5, TmMADS-7, TmMADS-9, TmMADS-14, TmMADS-17, TmMADS-23, TmMADS-29, TmMADS-32, TmMADS-36, TmMADS-37, TmMADS-38, TmMADS-43, TmMADS-44, TmMADS-49*, and *TmMADS-56*) (Figure 6B and Table 1). Some genes, such as *TkMADS-48*, *TkMADS-59*, *TmMADS-14*, and *TmMADS-44*, were involved in multiple duplication events.

Furthermore, synteny analysis indicated that *TkMADS* and *TmMADS* family members showed good collinearity, involving a total of 64 gene pairs (Figure 6C and Table 2). Among the *TkMADS* genes, 22 were homologous to only one *TmMADS* gene each, and 7 *TkMADS* genes were homologous to two or more *TmMADS* genes. Five *TkMADS* genes were homologous only to non-*TmMADS* genes in the TM genome, and three *TmMADS* genes were homologous only to non-*TkMADS* genes in the TKS genome. Nineteen *TkMADS* genes (*TkMADS-2*, *TkMADS-3*, *TkMADS-4*, *TkMADS-14*, *TkMADS-18*, *TkMADS-21*, *TkMADS-22*, *TkMADS-27*, *TkMADS-31*, *TkMADS-32*, *TkMADS-33*, *TkMADS-34*, *TkMADS-36, TkMADS-37, TkMADS-38*, *TkMADS-47*, *TkMADS-50*, *TkMADS-54*, and *TkMADS-66*) had no homologous genes in the TM genome (Supplemental Table S4), and these genes were specific to the TKS genome. Moreover, twenty *TmMADS* genes (33.9%) had no homologous genes in the TKS genome, and these genes were unique to the TM genome. In addition, there were 7135 collinear genes involving 4253 collinear chromosomal regions in TKS, accounting for 15.78% of the total genes. In the TM genome, there were 5617 collinear genes (accounting for 12.33% of the total genes) involving 3290 collinear chromosomal regions. Interestingly, *TkMADS* and *TmMADS* genes were located within these collinear regions (Figure 6C). These results suggested that most *TkMADS* and *TmMADS* genes were derived from the duplication of chromosomal regions, and that the expansion of *TkMADS* and *TmMADS* members was promoted by fragment duplication events.

### 2.6. Expression Profiles of TkMADS in Different Tissues of TKS

**Figure 7:**
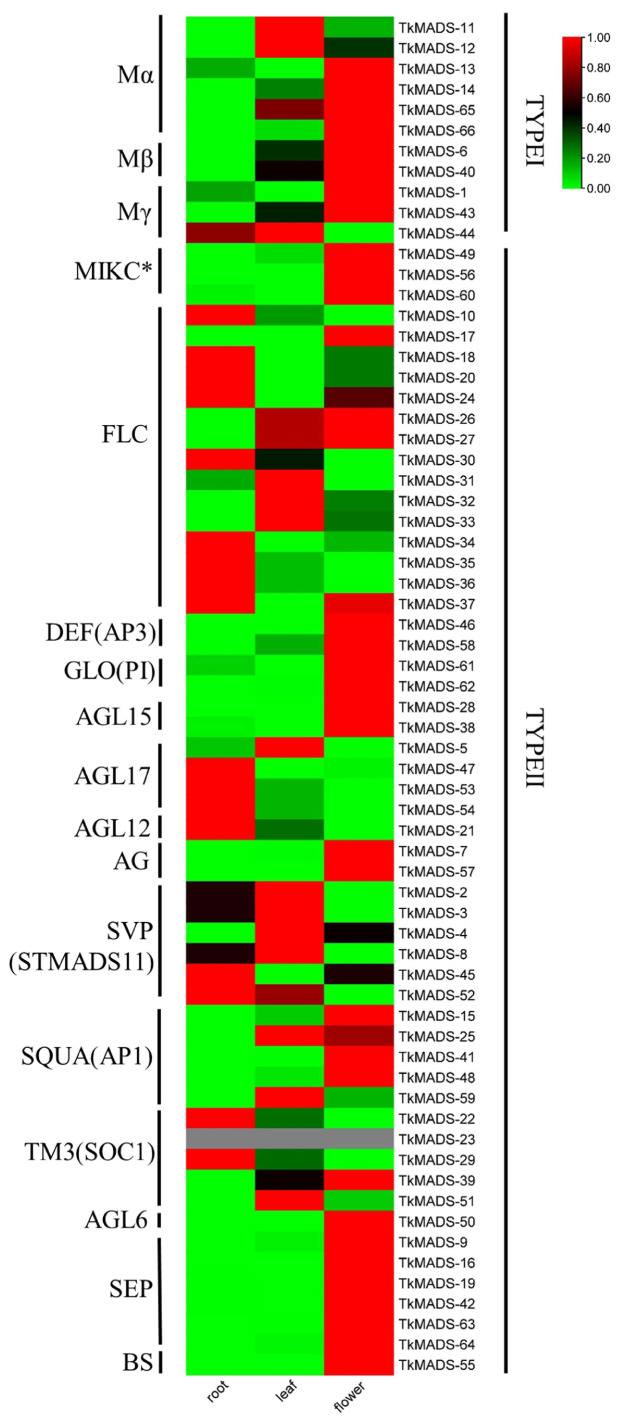
Expression profiles of *TkMADS* genes in different tissues of TKS. The expression levels of *TkMADS* genes were determined via qRT-PCR. *TkActin* was used as an internal standard. The gray bar indicates that no expression was detected, while the red, black, and green bars represent high, moderate, and low expression levels, respectively.

To identify the *TkMADS* genes involved in specific tissue development, the expression patterns of 66 *TkMADS* genes were analyzed in different tissues of TKS via qRT-PCR. As shown in Figure 7, the results indicated that most *TkMADS*s were expressed in all organs of TKS but exhibited tissue-specific expression patterns. In total, 35 genes (53%), namely, 8 type I genes (73%) and 27 type II genes (49%), were preferentially expressed in the flowers, suggesting that *TkMADS* genes have important roles involving reproductive growth in TKS. Similar expression patterns were detected for genes belonging to the same subclade, especially genes associated with the ABCDE model, suggesting that their functions are conserved and redundant after duplication. Except for *TkMADS-59* (AP1), all genes associated with the ABCDE model were preferentially expressed in the flowers, including the A-class genes *TkMADS-15*, *TkMADS-25 TkMADS-41*, and *TkMADS-48* (AP1); B-class genes *TkMADS-61* and *TkMADS-62* (PI), *TkMADS-46* and *TkMADS-58* (AP3); C-class genes *TkMADS-7* and *TkMADS-57* (AG); and the E-class genes *TkMADS-9*, *TkMADS-16*, *TkMADS-19*, *TkMADS-42*, *TkMADS-64*, and *TkMADS-63* (SEP). A total of 17 *TkMADS*s (all type II) were preferentially expressed in the roots, with expression levels more than twice those in the leaves or flowers (Figure 7). Among the expression levels of these genes, that of *TkMADS-52* was particularly high in the roots, and the level was 60 times higher than that in the flowers. These results suggested that these *TkMADS* genes may play an important role in root morphogenesis. Fourteen genes (namely, 3 type I genes and 11 type II genes) were preferentially expressed in the leaves. However, no *TkMADS-23* transcripts were detected in any of the analyzed tissues. Genes belonging to the same subfamily did not always exhibit the same expression patterns; this was true for some genes in the Mα and Mγ subfamilies (type I) and some genes in the FLC, SVP, AGL17, AGL12, and TM3 subfamilies (type II).

### 2.7. Identification of TkMADS Genes Related to Biomass Formation in TKS

**Figure 8:**
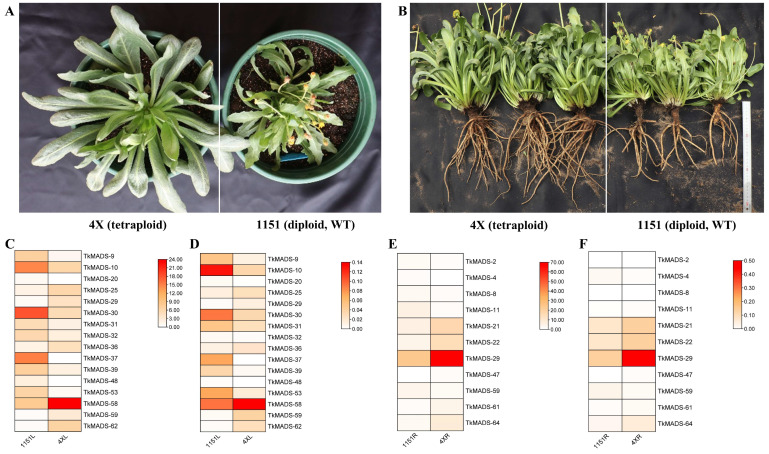
Expression of *TkMADS* genes related to biomass formation in tetraploid and diploid TKS. Phenotypes of tetraploid 4X and diploid 1151 plants cultured in a growth chamber (**A**) and non–temperature-controlled greenhouses (**B**) for 6 months. Differentially expressed *TkMADS* genes were identified by comparisons between the RNA-seq data of 4XL and 1151L (**C**) and between those of 4XR and 1151R (**E**) and further verified via qRT-PCR (**D**,**F**).

To increase the biomass of TKS, tetraploid TKS 4X plants were generated via oryzalin treatment [32]. Compared with those of wild-type (WT) TKS 1151 (a diploid), the leaf and root biomass of the tetraploid 4X plants significantly increased under different planting conditions (Figure 8A,B). Comparison of the 6-month-old plants showed that the leaf length and leaf width of tetraploid 4X increased by 72.4% and 95.8%, respectively. The number of lateral roots of 4X was 2.4 times that of WT 1151, and the taproot diameter was increased by 40.9%. Compared with that of the WT, the fresh root weight of 4X increased by 19.6%. In terms of NR content, there was no significant difference between the WT and tetraploid plants (Table 3). Although the NR content of the tetraploid 4X did not improve, a significant increase in the root biomass will increase the overall NR yield. To identify MADS-box genes associated with biomass formation in TKS, the leaf and root transcriptomic data of tetraploid TKS (4X, large biomass) and wild-type diploid TKS (1151, small biomass) plants were compared. A total of 39,468,474 (1151L), 41,527,124 (1151R), 42,506,920 (4XL), and 40,065,188 (4XR) clean reads were generated, of which more than 88% reads mapped to the TKS reference genome (GWH; http://bigd.big.ac.cn/gwh/, accessed on 21 December 2021) [28] (Supplemental Table S6). After functional annotation, 44 and 45 *TkMADS*s were expressed in the leaves and roots, respectively, of tetraploid 4X plants, while 43 and 44 *TkMADS*s were expressed in the leaves and roots, respectively, of diploid 1151 plants (Supplemental Table S7). Furthermore, comparative analysis of the leaf transcriptomes of 4XL vs. 1151L revealed that 16 *TkMADS* were differentially expressed, with 6 upregulated genes and 10 downregulated genes detected in 4XL (Figure 8C). A comparison of the root transcriptomes of 4XR vs. 1151R indicated that 6 *TkMADSs* were upregulated in 4XR and that 5 *TkMADSs* were downregulated in 4XR (Figure 8E). Moreover, the relative expression levels of these differentially expressed genes (DEGs) were verified via qRT-PCR (Figure 8D,F), and the results were consistent with those of the RNA-seq data. Among these DEGs, *TkMADS-29* was upregulated in both 4XL and 4XR, with expression levels being 6.2-fold and 2.8-fold those in 1151L and 1151R, respectively. The expression of *TkMADS-59* was upregulated in 4XL but downregulated in 4XR. Taken together, these results suggested that the differentially expressed *TkMADSs* identified in tetraploid TKS may be important transcription factors that regulate biomass formation in TKS.

### 2.8. Identification of TkMADS Genes Related to NR Biosynthesis in TKS

**Figure 9:**
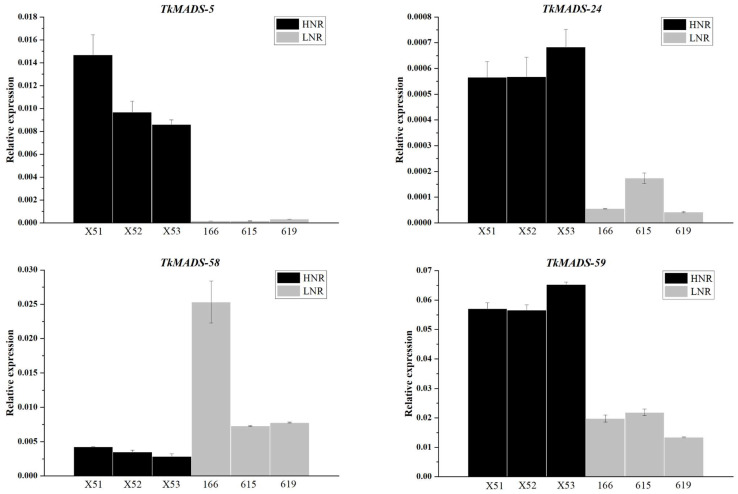
Expression of *TkMADS* genes related to NR biosynthesis in different lines of TKS verified via qRT-PCR. The high NR content (HNR) group includes X51, X52, and X53, and the low NR content (LNR) group includes 166, 615, and 619.

To explore the MADS-box genes related to NR biosynthesis in TKS roots, the root transcriptomic data of different high-NR content (HNR) lines (i.e., X51, X52, and X53) and low-NR content (LNR) lines (i.e., 166, 615, and 619) [33] were systematically analyzed. The results showed that 22 *TkMADS* genes were expressed in the HNR group, and 23 *TkMADS* genes were expressed in the LNR group (Supplemental Table S8). Moreover, comparative analysis of the root transcriptomes of HNR vs. LNR revealed that four *TkMADSs* (*TkMADS-5*, *TkMADS-24*, *TkMADS-58*, and *TkMADS-59*) were significantly differentially expressed in the HNR group. The expression abundances of *TkMADS-5*, *TkMADS-24*, and *TkMADS-59* in the HNR group were significantly higher than those in the LNR group, whereas the expression of *TkMADS-58* was significantly lower than that in the LNR group (Supplemental Table S8). Furthermore, the relative expression levels of these differentially expressed *TkMADS* genes in the roots of the plants in the HNR and LNR groups were verified via qRT-PCR (Figure 9), the results of which were consistent with the results of RNA-seq. The average expression levels of *TkMADS-5, TkMADS-24*, and *TkMADS-59* in the three HNR lines were 50.7, 6.7, and 3.2 times those in the LNR lines, respectively. However, the average expression level of *TkMADS-58* in the three HNR lines was only 26.2% of that in the LNR lines (Figure 9). The correlation analysis between the NR content of the six TKS lines and the expression levels of these four *TkMADS* genes demonstrated that the expression of *TkMADS-5, TkMADS-24*, and *TkMADS-59* was positively correlated with the NR content in TKS roots (*p* < 0.01) and that *TkMADS-58* expression was negatively correlated with the NR content (*p* < 0.01) (Table 4). Taken together, these results suggested that *TkMADS-5, TkMADS-24, TkMADS-58*, and *TkMADS-59* may be important transcription factors in regulating NR biosynthesis in TKS.

## 3. Discussion

Plant MADS-box genes regulate diverse biological processes, including vegetative growth and reproductive development, especially processes related to the development of inflorescences, flowers, and fruits [34,35]. The number of MADS-box genes varies greatly among different species. The retention of duplicate genes differs across species, leading to different numbers of MADS-box genes among different species, with different evolutionary constraints [36]. There are 109 MADS-box gene members in *Arabidopsis* [6], 82 members in lettuce [37], and 108 members in chrysanthemum [8]. A recently published article revealed that there are 78, 54, and 57 MADS-box genes in the diploid *Taraxacum officinale* (TO, 2n = 2x = 16), TM, and TKS genomes, respectively [38]. The large expansion in the number of MADS-box genes in TO may be due to the fact that its genome has not been able to assemble at the chromosomal level, which has 4059 scaffolds with an N50 size of 757 kb [38]. In this study, 66 *TkMADS* and 59 *TmMADS* genes were identified in the TKS and TM genomes, respectively. Compared to the recent article [38], there were more than 9 and 5 members in the TKS and TM genomes, respectively. The difference in the gene number may be due to the different parameters used in searching the TKS and TM genomes, and the use of transcriptome data from several samples completed in our lab. *Taraxacum* MADS-box genes identified in the TKS and TM genomes, and *Arabidopsis* MADS-box genes can be classified into two types (type I and type II) (Figure 1), and type I and type II members can be divided into 3 and 14 subfamilies, respectively (Figure 2). The *TkMADS* gene family members were present in all 17 subfamilies, and *TmMADS* members were present in 16 subfamilies (all but the BS subfamily) (Figure 2B). Comparing the MADS-box gene family in the three species, we found that there were fewer type I members in TKS and TM than in *Arabidopsis*. However, unlike in *Arabidopsis*, gene expansion was commonly found for the type II *TkMADS* and *TmMADS* genes. These findings were similar to those in TO [38], chrysanthemum [8], and lettuce [37], and these species together with TKS and TM belong to the Asteraceae family. We speculated that the reduction in type I genes and the expansion of type II genes in *Taraxacum* may contribute to the adaptability of *Taraxacum* plants in response to different environmental conditions. In particular, the FLC subgroup, consisting of 15 *TkMADS* and 8 *TmMADS* members, showed a large degree of gene expansion in *Taraxacum*, whereas there were only 6 in *Arabidopsis* (Figure 2B). *FLC* acts as a convergence point for environmental and endogenous pathways that regulate flowering time in *Arabidopsis* [34]. *FLC* has been identified as a key component of the response of some *Arabidopsis* ecotypes to vernalization [39]. Genes of the FLC subfamily were also found to have significantly expanded in wheat compared with *Arabidopsis* and rice [5]. However, rice [7] and watermelon [9], whose flowering does not require vernalization, have lost the FLC subfamily. Therefore, the expansion of *FLC* genes in vernalizing plants occurred as an adaptation to environmental conditions such as light and temperature in different regions.

Previous studies have suggested that an intron-rich gene would lose multiple introns simultaneously by retrotransposition, thereby producing intron-less ancestral genes. In this study, we found that type I *TkMADS* and *TmMADS* genes usually have no introns or only one intron (Figure 3), which suggests that the loss of multiple introns may have occurred during MADS-box gene family diversification. This phenomenon may be due to the diversity in the origin of retrotransposons or differences in the ancestry of the type I genes in terms of gaining or losing introns [7]. The distribution of introns in type II genes varies widely. Similar findings have also been reported in *Arabidopsis* and rice MADS-box genes [7,40], suggesting the evolutionary conservation of type I MADS-box genes among different plant species. However, some homologous genes showed different intron/exon arrangements, indicating that a more complex gene structural evolution may have occurred for *Taraxacum* MADS-box genes. Our conserved motif analysis indicated that the same groups of genes contain similar conserved motifs (Figure 4). These results suggested that these conserved motifs may play crucial roles in maintaining the conserved functions of MADS-box genes; this is particularly true for the K-box motif, which is a domain that functions in protein–protein interactions between MADS-box proteins and is present only in the type II proteins. However, high structural divergence was also found among different subgroups. Analyses of the gene structures and conserved motifs could provide more clues about the evolutionary relationships of the MADS-box genes in the TKS and TM genomes.

Gene duplication (segmental duplication and tandem duplication) as well as transposition events are prevalent forces that result in the expansion of family members and increased genome complexity in eukaryotes [31]. The duplication of more than two genes located on the same chromosome is evidence of tandem duplication events, whereas gene duplication that occurs on different chromosomes is identified as segmental duplication [30,41]. Both tandem duplication and segmental duplication have played crucial roles in MADS-box gene expansion in the genomes of TKS and TM. In this study, *TkMADS* and *TmMADS* genes were distributed on all 8 chromosomes of *Taraxacum*, but they were not evenly distributed on each chromosome. Chromosome 3 in both TKS and TM contained the largest number of MADS-box genes, with 23 *TkMADS* and 14 *TmMADS* genes, respectively. At the same time, MADS-box gene clusters were present at the distal ends of chromosomes 3 and 7 in TKS and TM (Figure 5). Notably, genes of the FLC subfamily were concentrated on chromosome 3 in TKS and TM, suggesting that tandem duplication mechanisms have driven the expansion of the members of this gene family. Moreover, the functional relevance of *TkMADS* and *TmMADS* genes formed tandem arrays on chromosomes, which may promote the evolution of new functions.

Synteny analysis revealed that most of the *TkMADS* and *TmMADS* genes showed good collinearity (Figure 6), indicating high homology between the TKS and TM genomes. Moreover, differentiation of some specific gene members was observed. For example, the BS subfamily of MADS-box genes was absent in the TM genome, but BS members were present in the TKS genome (*TkMADS-55*) (Figure 1). The BS subfamily of MADS-box genes plays a conserved role in ovule and fruit development [42,43]. Although TKS and TM are closely related *Taraxacum* species, their reproduction modes are completely different. TM undergoes apomixis, while TKS undergoes self-incompatibility. *TkMADS-55* of the BS subfamily was preferentially expressed in the flowers (Figure 7), suggesting that it may be related to the self-incompatibility of TKS.

Although TKS is a promising NR-producing plant, there are several disadvantages in need of overcoming for the commercialization process, such as self-incompatibility, low root biomass, and low NR content. To identify MADS-box genes that regulate the development of flowers and roots, *TkMADS* expression profiles were examined in different tissues of TKS via qRT-PCR in this study. All 66 *TkMADS*s showed different expression patterns (Figure 7), which provides insight for future studies on the functions of MADS-box genes in TKS growth and development. Type II (MIKC) MADS-box genes participate in almost every aspect of *Arabidopsis* plant development and constitute an important family of overall conserved genes [2,3,44,45]. In our study, more than half of the *TkMADS* members were preferentially expressed in the flowers, which confirmed their important roles in the reproduction of TKS. According to the ABCDE model, combinations of MADS-box proteins of different classes determine flower development and floral organ identity [21]. In the present study, genes associated with the ABCDE model were identified in TKS and its related species TM. Moreover, high and preferential expression of these genes in TKS flowers demonstrated their crucial roles in flower development (Figure 7). These genes will be targets for reproductive improvement and domestication of TKS.

In addition to flower development, increasing numbers of studies have shown that MADS-box genes play important roles in plant root development and morphogenesis [46]. In *Arabidopsis*, it was shown that nearly 50% of MIKC genes were expressed in the roots [6]. Moreover, several genes belonging to the AG, SOC1/AGL20, ANR1/AGL44, and AGL18 subfamilies were strongly and preferentially expressed in the roots [47]. *AGL12* is mostly expressed in the root meristem, particularly in the phloem cells of the vascular tissue, and it is also expressed in atrichoblasts of the root epidermis [48,49]. The AGL17 subfamily is involved in various functions, including root development, flowering time control, tillering, stomatal development, and stress response [50,51,52,53,54]. *AGL21*, another member of the AGL17 clade [55], is also highly expressed in the vascular tissue and in the stem cell niche of the primary root, including the quiescent center [6,56]. In this paper, nearly 33% of MIKC genes were preferentially expressed in the roots of TKS, including *TkMADS-10*, *TkMADS-18*, *TkMADS-20*, *TkMADS-24*, *TkMADS-30*, *TkMADS-34*, *TkMADS-35*, *TkMADS-36*, *TkMADS-37* (FLC subfamily), *TkMADS-45*, *TkMADS-52* (SVP subfamily), *TkMADS-47*, *TkMADS-53*, *TkMADS-54* (AGL17 subfamily), *TkMADS-21* (AGL12 subfamily), *TkMADS-22,* and *TkMADS-29* (SOC1 subfamily) (Figure 7). Moreover, comparing the root transcriptomes of 4XR vs. 1151R, we found that *TkMADS-21* (AGL12 subfamily), *TkMADS-22*, *TkMADS-29* (SOC1 subfamily), *TkMADS-47* (AGL17 subfamily)*, TkMADS-61* (GLO/PI subfamily), and *TkMADS-64* (AGL6 subfamily) were upregulated in 4XR, whereas *TkMADS-2*, *TkMADS-4*, *TkMADS-8* (SVP subfamily), *TkMADS-11* (Mα subfamily), *TkMADS-59* (AP1/SQUA subfamily) was downregulated in 4XR (Figure 8D,F). Further study of its functions in root growth and development will be helpful for increasing the root biomass of TKS via transgenic and gene editing technologies.

To better understand the role of *TkMADS* genes involved in NR biosynthesis, we identified four *TkMADSs* (*TkMADS-5, TkMADS-24, TkMADS-58,* and *TkMADS-59*) that were differentially expressed between the roots of high-NR content lines and those of the low-NR content lines (Figure 9). Interestingly, the expression of *TkMADS-5*, *TkMADS-24*, and *TkMADS-59* was positively correlated with NR content in TKS roots (*p* < 0.01), while *TkMADS-58* expression was negatively correlated with NR content (*p* < 0.01) (Table 4). These four genes, *TkMADS-5* (AGL17 subfamily), *TkMADS-24* (FLC subfamily), *TkMADS-58* (DEF subfamily), and *TkMADS-59* (AP1/SQUA subfamily), were from different subfamilies (Figure 1). Members of the DEF subfamily are generally expressed in the flowers [57], the FLC subfamily members comprise a class of flowering inhibitors [58], the AP1/SQUA subfamily members are generally expressed in the flowers and fruits [59,60], and the AGL17 subfamily members are mainly expressed in the roots [46]. We speculated that the *TkMADS*s of these subfamilies have evolved new functions in TKS. Here, we provide new targets for future breeding efforts to improve TKS performance, especially in enhancing root biomass and NR production.

## 4. Materials and Methods

### 4.1. Plant Material and Growth Conditions

Tissue culture-generated seedlings of TKS lines with low-NR content (166, 615, and 619 with 2.09%, 3.09%, and 3.27% NR content, respectively), lines with high NR content (X51, X52, and X53 with 9.21%, 8.92%, and 9.06% NR content, respectively), 1151 (wild-type TKS, which is diploid and produces a low amount of biomass), tetraploid TKS 4X (which produces a large amount of biomass and is induced by oryzalin) [32], and EB1 (which flowers early) were used in this study. The plants were grown in a plant growth chamber (MMM/Climacell 707, Planegg, Germany) set at 23 °C with a 16 h light/8 h dark photoperiod (light intensity of 13,000 lux). At the flowering stage, samples of leaves, roots, and flowers were collected from EB1 plants simultaneously and used for expression analysis of *TkMADS* genes in different tissues. In addition, the fresh leaves and roots of four-month-old seedlings of 1151, 4X, 166, 615, 619, X51, X52, and X53 were collected and subjected to RNA sequencing (RNA-seq) and qRT-PCR analysis.

### 4.2. Identification of MADS-Box Genes in the TKS and TM Genomes

Local TKS and TM genome and transcriptome databases were established using the published genomic data of TKS and TM [28]. To identify MADS-box genes in TKS and TM, two strategies were used: (1) the hidden Markov model (HMM) profiles of the MADS domain (PF00319) and the K-box domain (PF01486) were used as seed files to search the protein databases for TKS and TM protein sequences via HMMER 3.0 [61] with an E-value threshold of 0.05, and (2) MADS protein sequences from *Arabidopsis thaliana* (http://www.ncbi.nlm.nih.gov/genome/4?genome_assembly_id=380024, TAIR10, accessed on 11 July 2019) were used as queries for BLASTP searches against TKS and TM protein sequences with an E-value cutoff of 0.05. Gene sequences with more than 99% identity or at the same physical location were considered redundant sequences and were removed to obtain candidate *TkMADS* and *TmMADS* genes. To validate the accuracy of these candidate *TkMADS* and *TmMADS* genes, the Simple Modular Architecture Research Tool (SMART) 4.0 was used to confirm the completeness of the MADS-box domain. The online tool SMS2 (http://www.detaibio.com/sms2/protein_iep.html, accessed on 27 August 2015) was employed to analyze the CG content of *TkMADS* and *TmMADS* sequences, determine the theoretical isoelectric point (PI), and determine molecular weight (MW) of the encoded proteins.

### 4.3. Phylogenetic Analysis of TkMADS and TmMADS Proteins

To understand the phylogenetic relationship and classification of *TkMADS* and *TmMADS* genes, individual unrooted NJ trees were constructed based on the full-length amino acid sequences of MADS-box genes from *Arabidopsis thaliana* (http://www.ncbi.nlm.nih.gov/genome/4?genome_assembly_id=380024, TAIR10, accessed on 11 July 2019), TKS, and TM according to reported methods [62,63,64]. The MADS-box protein sequences were subsequently aligned via ClustalW. Phylogenetic trees were constructed via MEGA X software 10.2.2. All the neighbor-joining (NJ) tree nodes were evaluated via bootstrap analysis with 1000 replicates, an LG+G model, missing data treatment set to partial deletion, and site coverage cutoff set to 60%. Branches with bootstrap values under 50% were collapsed.

### 4.4. Analysis of Gene Structures and Conserved Motifs of Encoded Proteins

The CDS and corresponding genomic DNA sequences of *TkMADS* and *TmMADS* genes were retrieved from the TKS and TM genome sequences to predict gene structure. The online tool GSDS 2.0 (http://gsds.cbi.pku.edu.cn/index.php, accessed on 10 December 2014) was used to determine the exon–intron structure [65]. Conserved motifs of TkMADS and TmMADS proteins were identified via online software MEME 4.12.0 (http://meme-suite.org/tools/meme, accessed on 5 February 2023), with the following parameters: 10 different motifs, motif width of 6–200 amino acids, and any number of repetitions. The SMART database was used to annotate the motifs identified via MEME.

### 4.5. Chromosomal Localization, Duplication, and Collinearity Analysis of the TkMADS and TmMADS Genes

All the *TkMADS* and *TmMADS* genes were mapped to the chromosomes with MapChart software 2.32, according to their positions in the TKS and TM genomic databases, respectively. Multiple Collinearity Scan Toolkit (MCScanX, Athens, Greece) [66] was employed to analyze the duplication events for each *TkMADS* and *TmMADS* gene with the default parameters used [67].

### 4.6. Expression Profiling of TkMADS Genes in Different Tissues

To reveal the expression patterns of the *TkMADS* genes involved in TKS growth and development, total RNA was extracted from the roots, leaves, and flowers with the RNAprep Pure Plant Plus Kit (Polysaccharides & Polyphenolics-rich) (TIANGEN Biotech, Beijing, China) according to the recommended method of the manufacturer. Then the extracted RNA was treated with RNase-free DNase I to remove genomic DNA contamination. The quality and quantity of the extracted RNA were checked via agarose gel electrophoresis and the values of A260/A280 (A260/A280 between 1.8 and 2.1) were measured with a spectrophotometer (Implen P-330-31-10, Munich, Germany). cDNA was synthesized with oligo (dT)20 primers using M-MLV reverse transcriptase (Promega, Madison, WI, USA) using 1 µg total RNA as the template. For quantification of the relative expression levels of *TkMADS*, qRT-PCR analysis was performed with a CFX96 Real-Time System (Bio-Rad, Hercules, CA, USA) using the GoTaq qPCR Master Mix (Promega, Madison, WI, USA) according to the manufacturer’s instructions. The PCR was performed in a 20 μL reaction mixture containing 1×GoTaq qPCR Master Mix, 0.2 μM each primer, and approximately 30 ng of cDNA per sample. The reaction program was as follows: 2 min at 95 °C for denaturation, followed by 40 cycles of 10 s at 95 °C and 30 s at 60 °C. Then raw data acquisition and analysis were performed using CFX Maestro software 2.3. The *TkActin* gene (GenBank accession: DY824357) was used as an internal standard, and the 2^−∆∆CT^ method was used to calculate the relative expression levels of target genes [68]. All the experiments were performed for three biological triplicates with three technical replicates. All the primers used were designed with Primer 5.0 software and are shown in Supplemental Table S5.

### 4.7. Identification of TkMADS Genes Related to the Biomass Formation and NR Biosynthesis in TKS

The leaf and root samples of the tetraploid TKS 4X (large biomass) and wild-type diploid TKS 1151 (low biomass) were subjected to RNA-seq according to a cBot Cluster Generation System on an Illumina platform at Biomarker Technology Company (Beijing, China). RNA concentration and purity was measured using NanoDrop 2000 (Thermo Fisher Scientific, Wilmington, DE, USA). RNA integrity was assessed using the RNA Nano 6000 Assay Kit of the Agilent Bioanalyzer 2100 system (Agilent Technologies, Santa Clara, CA, USA). A total amount of 1 μg RNA per sample was used as input material for the RNA sample preparations. Sequencing libraries were generated using NEBNext UltraTM RNA Library Prep Kit for Illumina (NEB, Ipswich, MA, USA) following the manufacturer’s recommendations and index codes were added to attribute sequences to each sample. Then clustering of the index-coded samples was performed on a cBot Cluster Generation System using TruSeq PE Cluster Kit v4-cBot-HS (Illumia, San Diego, CA, USA) according to the manufacturer’s instructions. After cluster generation, the library preparations were sequenced on an Illumina platform and paired-end reads were generated. Raw data (raw reads) of fastq format were firstly processed through in-house perl scripts. In this step, clean data (clean reads) were obtained by removing reads containing adapter, reads containing ploy-N, and low quality reads from raw data. At the same time, Q20, Q30, GC-content, and sequence duplication level of the clean data were calculated. All the downstream analyses were based on clean data with high quality. The adaptor sequences and low-quality sequence reads were removed from the data sets. Raw sequences were transformed into clean reads after data processing. These clean reads were then mapped to the TKS reference genome (GWH; http://bigd.big.ac.cn/gwh/, accessed on 21 December 2021) [28] using the Hisat2 tools. Only reads with a perfect match or one mismatch were further analyzed and annotated based on the reference genome. The statistics of raw and clean reads of each sample are listed in Supplemental Table S6.

The root samples of different TKS lines with low NR content (166, 615 and 619), and lines with high NR content (X51, X52 and X53) were subjected to RNA-seq according to the Oxford Nanopore Technologies (ONT) protocol on the PromethION platform at Biomarker Technology Company (Beijing, China), which was described in detail in our published article [33]. The raw and clean reads of each sample, and data processing were performed as described in our previous study [33]. Briefly, raw reads were filtered with minimum average read quality score = 7 and minimum read length = 500 bp, and the ribosomal RNAs were discarded. Then, full-length, non-chimeric (FLNC) transcripts were identified by determining the primers at both ends of the reads. Finally, clusters of the FLNC transcripts were obtained for each sample after mapping them to the TKS reference genome (GWH; http://bigd.big.ac.cn/gwh/, accessed on 21 December 2021) with mimimap2 [28]. The mapped reads were further collapsed via the cDNA_Cupcake package with min-coverage = 85% and min-identity = 90%, and the 5′ difference was not considered.

Differential expression analysis of two groups was performed using the DESeq2. Genes with a false discovery rate (FDR) < 0.01 and a fold-change ≥2 according to DESeq were considered DEGs. To identify *TkMADS* genes related to biomass formation in TKS, comparative analysis of the 4XL vs. 1151L leaf transcriptomes and 4XR vs. 1151R root transcriptomes was conducted. The TKS root transcriptomic databases derived from different TKS lines with low and high NR contents were used to identify *TkMADS* genes related to NR content [33]. The transcript abundance was evaluated using fragments per kilobase of exon model per million mapped fragments (FPKM) or the counts per million or counts of exon model per million mapped reads (CPM) (Supplemental Tables S7 and S8). Heatmaps of gene transcript abundance were produced from the “heatmap” package in R.

### 4.8. Statistical Analysis

The data were analyzed with SPSS 24.0 (IBM Corporation, New York, NY, USA), and *p* < 0.05 was statistically significant. The correlation analysis was performed via the correlate tool for partial correlations and two-tailed test of significance, and NR content was set as the control and gene expression values were set as variables. The histograms were drawn with OriginPro 2018 (Origin Lab Corporation, Northampton, MA, USA).

## 5. Conclusions

In this study, a total of 66 *TkMADS* and 59 *TmMADS* were identified from the TKS and TM genomes, and their evolutionary mechanisms, conserved functions and differentiation of new functions were investigated. From the diploid TKS to triploid TM, the total number of MADS-box genes had not increased, but expansion occurred in specific subfamilies. The MADS-box genes in TKS and TM were highly conserved, and showed good collinearity. Most *TkMADS* genes of type II were highly expressed in the flowers, suggesting the conserved and dominant functions of MADS-box genes in flower development and self-incompatibility in TKS. Moreover, 25 *TkMADS* genes related to biomass formation, and 4 *TkMADS* genes related to NR content in TKS were identified, which could serve as new targets for improving NR yield through transgenic and gene editing technologies. The *TkMADS* and *TmMADS* gene families identified in this study provide important information for further elucidating the functions of MADS-box genes in *Taraxacum* species, and hastening the domestication of TKS.

## Figures and Tables

**Table 1:** Duplications of MADS-box genes in the genomes of TKS and TM.

Chr.	Subfamily	Duplicate Gene Pair		Subfamily	Chr.
Chr0	SVP(STMADS11)	*TkMADS-2*	*TkMADS-8*	SVP(STMADS11)	Chr2
Chr2	SEP	*TkMADS-9*	*TkMADS-16*	SEP	Chr3
Chr2		*TkA02G497120.1*	*TkMADS-48*	SQUA(AP1)	Chr6
Chr2	Mα	*TkMADS-11*	*TkMADS-65*	Mα	Chr8
Chr3	FLC	*TkMADS-17*	*TkMADS-20*	FLC	Chr3
Chr3	SEP	*TkMADS-19*	*TkMADS-42*	SEP	Chr6
Chr3	SQUA(AP1)	*TkMADS-15*	*TkMADS-48*	SQUA(AP1)	Chr6
Chr3	SQUA(AP1)	*TkMADS-25*	*TkMADS-59*	SQUA(AP1)	Chr7
Chr3	TM3(SOC1)	*TkMADS-23*	*TkMADS-51*	TM3(SOC1)	Chr7
Chr6	SQUA(AP1)	*TkMADS-48*	*TkMADS-59*	SQUA(AP1)	Chr7
Chr7	SQUA(AP1)	*TkMADS-59*	*TkMADS-63*	SEP	Chr8
Chr2	SEP	*TmMADS-7*	*TmMADS-14*	SEP	Chr3
Chr2	SVP(STMADS11)	*TmMADS-5*	*TmMADS-36*	SVP(STMADS11)	Chr6
Chr2	Mα	*TmMADS-9*	*TmMADS-56*	Mα	Chr8
Chr3	SEP	*TmMADS-14*	*TmMADS-38*	SQUA(AP1)	Chr6
Chr3		*TmA03G034970.2*	*TmMADS-32*	SEP	Chr6
Chr3		*TmA03G052240.1*	*TmMADS-37*	DEF(AP3)	Chr6
Chr3		*TmA03G052240.1*	*TmMADS-49*	DEF(AP3)	Chr7
Chr3	TM3(SOC1)	*TmMADS-23*	*TmMADS-44*	TM3(SOC1)	Chr7
Chr3	FLC	*TmMADS-17*	*TmA08G005080.1*		Chr8
Chr4	TM3(SOC1)	*TmMADS-29*	*TmA05G106130.1*		Chr5
Chr6	AGL6	*TmMADS-43*	*TmMADS-44*	TM3(SOC1)	Chr7

**Table 2:** Collinear genes between the TKS and TM genome.

TM Chr.	Subfamily	Collinear Genes Pair		Subfamily	TKS Chr.
Chr2	Mα	*TmMADS-9*	*TkMADS-65*	Mα	Chr8
Chr3	SQUA(AP1)	*TmMADS-18*	*TkMADS-63*	SEP	Chr8
Chr8	Mα	*TmMADS-51*	TkA08G096410.1		Chr8
Chr8	GLO(PI)	*TmMADS-53*	*TkMADS-61*	GLO(PI)	Chr8
Chr8		TmA08G002590.3	*TkMADS-62*	GLO(PI)	Chr8
Chr8		TmA08G005080.1	*TkMADS-63*	SEP	Chr8
Chr8	SEP	*TmMADS-54*	*TkMADS-64*	SEP	Chr8
Chr8	Mα	*TmMADS-56*	*TkMADS-65*	Mα	Chr8
Chr4	Mγ	*TmMADS-28*	*TkMADS-1*	Mγ	Chr0
Chr1	AGL17	*TmMADS-1*	*TkMADS-5*	AGL17	Chr1
Chr1	Mβ	*TmMADS-3*	*TkMADS-6*	Mβ	Chr1
Chr2	AG	*TmMADS-4*	*TkMADS-7*	AG	Chr2
Chr2		TmA02G114620.2	*TkMADS-10*	FLC	Chr2
Chr2	Mα	*TmMADS-9*	*TkMADS-11*	Mα	Chr2
Chr2	Mα	*TmMADS-10*	*TkMADS-12*	Mα	Chr2
Chr2		TmA02G122710.1	*TkMADS-13*	Mα	Chr2
Chr2	SEP	*TmMADS-7*	*TkMADS-9*	SEP	Chr2
Chr2	SQUA(AP1)	*TmMADS-8*	TkA02G497120.1		Chr2
Chr2	SVP(STMADS11)	*TmMADS-5*	*TkMADS-8*	SVP(STMADS11)	Chr2
Chr8	Mα	*TmMADS-56*	*TkMADS-11*	Mα	Chr2
Chr2	SEP	*TmMADS-7*	*TkMADS-15*	SQUA(AP1)	Chr3
Chr3	AG	*TmMADS-22*	TkA03G524020.3		Chr3
Chr3	TM3(SOC1)	*TmMADS-23*	*TkMADS-29*	TM3(SOC1)	Chr3
Chr3	FLC	*TmMADS-24*	*TkMADS-30*	FLC	Chr3
Chr3	FLC	*TmMADS-25*	*TkMADS-35*	FLC	Chr3
Chr3	FLC	*TmMADS-17*	*TkMADS-24*	FLC	Chr3
Chr3	SQUA(AP1)	*TmMADS-18*	*TkMADS-25*	SQUA(AP1)	Chr3
Chr3	FLC	*TmMADS-19*	*TkMADS-26*	FLC	Chr3
Chr3		TmA03G034970.2	*TkMADS-28*	AGL15	Chr3
Chr3	FLC	*TmMADS-15*	*TkMADS-20*	FLC	Chr3
Chr3	SEP	*TmMADS-14*	*TkMADS-16*	SEP	Chr3
Chr3	FLC	*TmMADS-15*	*TkMADS-17*	FLC	Chr3
Chr6	SEP	*TmMADS-32*	*TkMADS-19*	SEP	Chr3
Chr6	SQUA(AP1)	*TmMADS-38*	*TkMADS-15*	SQUA(AP1)	Chr3
Chr7	TM3(SOC1)	*TmMADS-44*	*TkMADS-23*	TM3(SOC1)	Chr3
Chr8		TmA08G005080.1	*TkMADS-24*	FLC	Chr3
Chr4	TM3(SOC1)	*TmMADS-29*	*TkMADS-39*	TM3(SOC1)	Chr4
Chr4	Mβ	*TmMADS-30*	*TkMADS-40*	Mβ	Chr4
Chr5	SQUA(AP1)	*TmMADS-31*	*TkMADS-41*	SQUA(AP1)	Chr5
Chr2	SEP	*TmMADS-7*	*TkMADS-48*	SQUA(AP1)	Chr6
Chr2	SVP(STMADS11)	*TmMADS-5*	*TkMADS-45*	SVP(STMADS11)	Chr6
Chr3	SEP	*TmMADS-14*	*TkMADS-48*	SQUA(AP1)	Chr6
Chr6	SVP(STMADS11)	*TmMADS-36*	*TkMADS-45*	SVP(STMADS11)	Chr6
Chr6	DEF(AP3)	*TmMADS-37*	*TkMADS-46*	DEF(AP3)	Chr6
Chr6	Mγ	*TmMADS-33*	*TkMADS-43*	Mγ	Chr6
Chr6	Mγ	*TmMADS-35*	*TkMADS-44*	Mγ	Chr6
Chr6	MIKC*	*TmMADS-42*	*TkMADS-49*	MIKC*	Chr6
Chr6	SEP	*TmMADS-32*	*TkMADS-42*	SEP	Chr6
Chr6	SQUA(AP1)	*TmMADS-38*	*TkMADS-48*	SQUA(AP1)	Chr6
Chr8		TmA08G005080.1	*TkMADS-48*	SQUA(AP1)	Chr6
Chr3		TmA03G052240.1	*TkMADS-58*	DEF(AP3)	Chr7
Chr3	SQUA(AP1)	*TmMADS-18*	*TkMADS-59*	SQUA(AP1)	Chr7
Chr3	TM3(SOC1)	*TmMADS-23*	*TkMADS-51*	TM3(SOC1)	Chr7
Chr6	AGL6	*TmMADS-43*	*TkMADS-51*	TM3(SOC1)	Chr7
Chr6	DEF(AP3)	*TmMADS-37*	*TkMADS-58*	DEF(AP3)	Chr7
Chr7	MIKC*	*TmMADS-47*	*TkMADS-55*	BS	Chr7
Chr7	MIKC*	*TmMADS-47*	*TkMADS-56*	MIKC*	Chr7
Chr7	AG	*TmMADS-48*	*TkMADS-57*	AG	Chr7
Chr7	DEF(AP3)	*TmMADS-49*	*TkMADS-58*	DEF(AP3)	Chr7
Chr7	MIKC*	*TmMADS-50*	*TkMADS-60*	MIKC*	Chr7
Chr7	TM3(SOC1)	*TmMADS-44*	*TkMADS-51*	TM3(SOC1)	Chr7
Chr7	SVP(STMADS11)	*TmMADS-45*	*TkMADS-52*	SVP(STMADS11)	Chr7
Chr7		TmA07G095680.1	*TkMADS-53*	AGL17	Chr7
Chr8		TmA08G005080.1	*TkMADS-59*	SQUA(AP1)	Chr7

**Table 3:** Comparison of characteristics of tetraploid TKS and its wild type.

Characteristics	Wild Type 1151	Tetraploid 4X
Leaf length	10.66 ± 0.37	18.37 ± 1.30 *
Leaf width	2.08 ± 0.01	4.07 ± 0.03 **
Number of lateral roots	8.00 ± 1.00	19.00 ± 1.73 **
Taproot diameter (cm)	2.53 ± 0.07	3.57 ± 0.34 *
Fresh root weight (g)	65.87 ± 1.15	78.76 ± 2.39 **
NR content (%)	3.82 ± 0.23	3.64 ± 0.22

* represents significant difference with *p* < 0.05; ** represents significant difference with *p* < 0.01.

**Table 4:** Correlation analysis of NR content and gene expression level.

Correlation Coefficient						
		NR Content	*TKMADS-5*	*TKMADS-24*	*TkMADS-58*	*TKMADS59*
Relevance	NR content	1.000				
	*TkMADS-5*	0.945 **	1.000			
	*TkMADS-24*	0.975 **	0.890 **	1.000		
	*TkMADS-58*	−0.724 **	−0.593 **	−0.686 **	1.000	
	*TkMADS-59*	0.971 **	0.901 **	0.994 **	−0.613 **	1.000

** represents significant difference with *p* < 0.01.

## Data Availability

Data and materials will be made available upon reasonable request.

## Supplementary Materials

The supporting information can be downloaded at: https://www.mdpi.com/article/10.3390/ijms241310997/s1.
